# A system dynamics modelling simulation based on a cohort of hepatitis B epidemic research in east China community

**DOI:** 10.1017/S0950268819000220

**Published:** 2019-02-28

**Authors:** Zhixin Yu, Min Deng, Chunting Peng, Xue Song, Yi Chen, Xue Zhang, Qiuxia Liu, Yuchuan Li, Haiyin Jiang, Xiaolan Xu, Liya Pan, Jing Yuan, Bing Ruan

**Affiliations:** 1 State Key Laboratory for Diagnosis and Treatment of Infectious Diseases, The First Affiliated Hospital, School of Medicine, Zhejiang University, Hangzhou, China; 2 The third people's hospital of Shenzhen, Zhejiang University, Shenzhen, Guangdong, China

**Keywords:** Cohort study, hepatitis B, system dynamics modelling

## Abstract

Hepatitis B constitutes a severe public health challenge in China. The Community-based Collaborative Innovation hepatitis B (CCI-HBV) project is a national epidemiological study of hepatitis B and has been conducting a comprehensive intervention in southern Zhejiang since 2009.

The comprehensive intervention in CCI-HBV areas includes the dynamic hepatitis B screening in local residents, the normalised treatment for hepatitis B infections and the upcoming full-aged hepatitis B vaccination. After two rounds of screening (each round taking for 4 years), the initial epidemiological baseline of hepatitis B in Qinggang was obtained, a coastal community in east China. By combining key data and system dynamics modelling, the regional hepatitis B epidemic in 20 years was predicted.

There were 1041 HBsAg positive cases out of 12 228 people in Round 1 indicating HBV prevalence of 8.5%. Of the 13 146 people tested in Round 2, 1171 people were HBsAg positive, with a prevalence of 8.9%. By comparing the two rounds of screening, the HBV incidence rate of 0.192 per 100 person-years was observed. By consulting electronic medical records, the HBV onset rate of 0.533 per 100 person-years was obtained. We generated a simulated model to replicate the real-world situation for the next two decades. To evaluate the effect of interventions on regional HBV prevalence, three comparative experiments were conducted.

In this study, the regional hepatitis B epidemic in 20 years was predicted and compared with HBV prevalence under different interventions. Owing to the existing challenges in research methodology, this study combined HBV field research and simulation to provide a system dynamics model with close-to-real key data to improve prediction accuracy. The simulation also provided a prompt guidance for the field implementation.

## Introduction

### HBV in China

The prevalence of hepatitis B varies greatly in different regions [1, 2], with the Asia-Pacific region having a high prevalence rate for hepatitis B [3, 4]. According to Chinese national HBVsero-epidemiological investigations in 2006, the estimated number of chronic HBV infections was 93 million in China [5]. The Chinese government has been conducting hepatitis B vaccination for infants since 1992. After years of efforts, the HBsAg prevalence rate in people aged under 20 years decreased significantly from over 10% (1992) [2] to under 2% (2006) [5]. Based on recent estimates, China has the highest HBV-infected population, but the number of HBV-infected children aged under 5-years-old in China ranks 10^th^ in the world [6]. Of the 16 countries with the highest number of HBV-infected children, China is the only country with a hepatitis B three-needle vaccination rate of more than 90% [6]. This also proves the effect of immune barrier conduction to control the prevalence of hepatitis B. However, HBsAg prevalence rate in the adult population remains relatively high ranging from 8% to 12% in China [5].

### Community-based Collaborative Innovation-HBV project

The Community-based Collaborative Innovation (CCI) project is a national epidemiological study on HIV/AIDS, tuberculosis and hepatitis B. Initiated in 2008, CCI-HBV project has established seven provincial-/city-level demonstration areas nationwide, covering more than 1 500 000 residents. After signing the health contract, the interventions consist of dynamic HBsAg screening for all residents, full-population vaccination for the uninfected people and a comprehensive normalised treatment for the patients including hepatitis B antiviral treatment (nucleoside analogues (NAs)) as well as symptomatic treatment [7, 8]. Electronic data were uploaded to the specific cloud information platform, laying a foundation for quality control and data analysis. Yuhuan County is one of the 12 county-level demonstration areas in Zhejiang Province. As a high prevalence area for hepatitis B in southeast China, Qinggang Town in Yuhuan County, where neonatal hepatitis B vaccination program has been implemented very well, was selected for further research.

### System dynamic modelling

The system dynamics method was created in the 1950s by MIT professor Jay Forrester. With his scientific and engineering background, Forrester attempted to use the laws of physics, especially circuit rules, to study economic and social systems. Today, system dynamics are often used in the long-term strategic models, which assume high levels of object aggregation [9, 10]. To predict and analyse the results of CCI HBV intervention, the high abstraction simulation level – system dynamics model was chosen for the evaluation. System dynamics model is suitable for integrating disease programs with prevention and control, especially for exploring the prevalence and spread of infectious diseases [11]. By combining local specific hepatitis B epidemiological features with system dynamics simulation, the prediction of the hepatitis B epidemiology in Qinggang in the next 20 years was realised.

## METHOD

### Cohort management and data source

The HBV dynamic screening in Qinggang Town is a part of the national hepatitis B demonstration project with a technical design based on standard operation procedures (registration number: country as Word −2013-L-00090505) [12]. In 2015, World Health Organisation proposed the overall target of international hepatitis B control through the eradication of viral hepatitis which will be a major threat to public health by 2030. To achieve this goal, CCI hepatitis B demonstration project was improved through multi-dimensional intervention, which included the dynamic hepatitis B screening, the normalised treatment for hepatitis B infection group and the upcoming hepatitis B vaccination for all age groups from 2019.

**Fig. 1.**
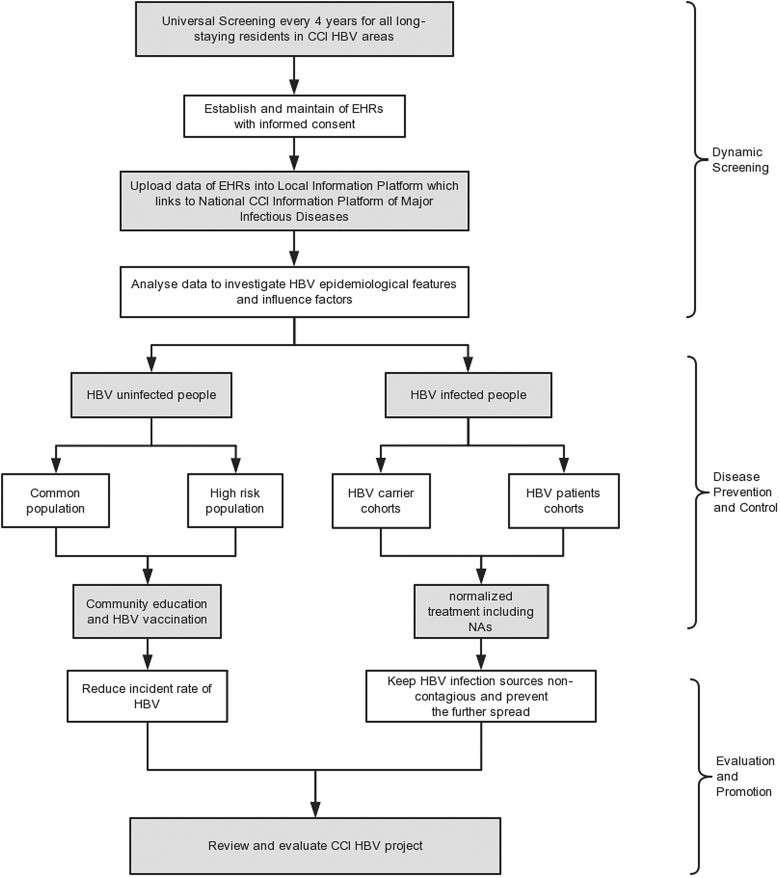
The flowchart of CCI HBV field research.

Figure 1 shows the flow chart of HBV dynamic screening and community management. To date, there are three community clinics (the Qinggang Village Community Health Service Station, the Broom Hill Community Health Service Station and the Third Community Health Service Station) and one healthcare centre (the Qinggang Town Healthcare Centre) in Qinggang town, with a total of 58 community doctors and general practitioners (GPs). Since 2009, community doctors in clinics encouraged and signed health service contracts with all long-stay residents in Qinggang (>6 months/year) with their informed consent. The GPs in health centre (teaming up with the community doctors in clinics) organised a centralised HBV-related screening including abdominal ultrasonography and serological tests of HBsAg, HBeAg, ALT, anti-HCV, serum level of ALT and AFP. After uploading the results in the corresponding electronic health record, community doctors provided health consultation for healthy people and designed the individualised treatment for hepatitis B patients in different stages. Because of the outflow of young people and the HBV infant vaccination program, this study focused on the hepatitis B transmission in the adult group. Considering the small number of local adults who have been actively vaccinated, the full-aged hepatitis B vaccination would be initiated from 2019. Our target is to establish a health screening system covering all residents as it needs to be further improved.

By December 2016, the related data had been uploaded to Infectious Disease Information Cloud Platform which was specifically built for CCI project data storage and analysis. With the informed consent of participates, this study was approved by the Human Research Ethics Committee of Zhejiang University (HREC reference: 2009 Ethical Review/Scientific Research, No. 4) on 30 July 2009. During the implementation, the subject's profiles were not used for any other irrelevant purposes and it was the responsibility of the Ethics Committee to oversee any other ethical issues.

### Model structure and data analysis

**Fig. 2.**
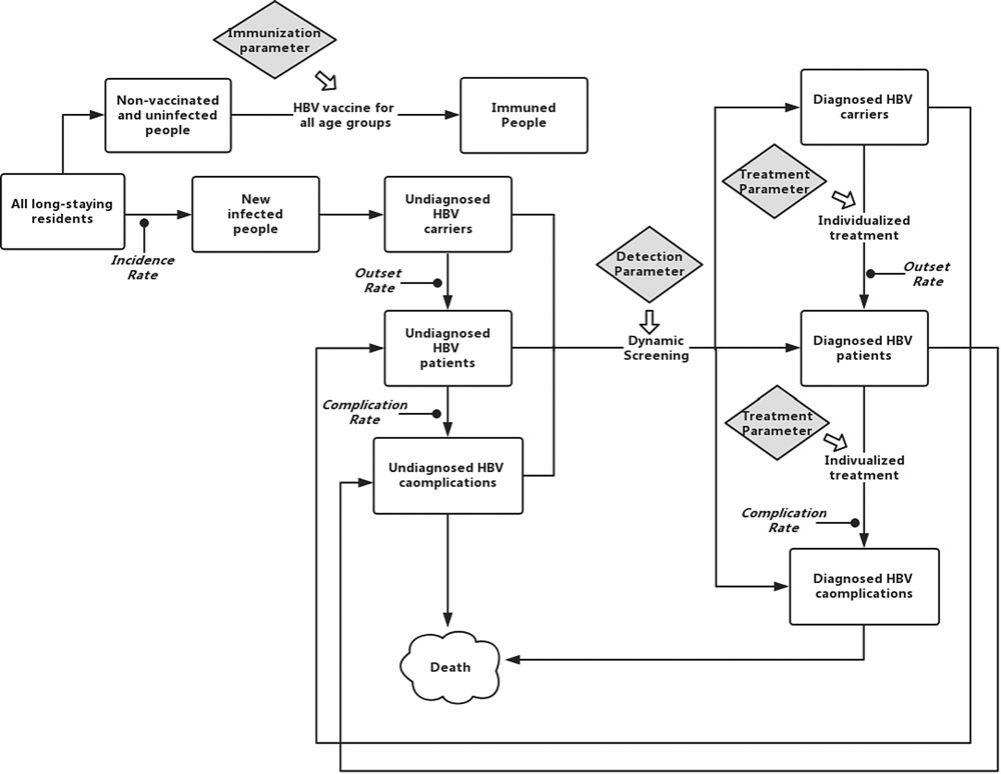
The overview of model structure, showing the population stocks and flows, including three parameters of community interventions.

The study had a lifetime horizon and held the perspective of a system dynamics model. The model structure (Fig. 2) was designed according to the natural history of hepatitis B and CCI HBV interventions. For the local population, the infected people were classified by two criteria – diagnosis status and disease progression. Thus, the residents were divided into uninfected people, immunised people, undiagnosed/diagnosed HBV carriers, undiagnosed/diagnosed HBV chronic patients and undiagnosed/diagnosed HBV complication patients.

According to the 2015 China Hepatitis B Prevention Guidelines, the prevalence of hepatitis B in different regions varies greatly. To simulate real event more precisely, the residents’ data in CCI HBV screening was analysed and the specific incidence rate and onset rate of hepatitis B epidemic in Qinggang Town was obtained. It was assumed that, the level of hepatitis B incidence rate and onset rate were constant over time and remained stable for the next 20 years. Due to the large differences in the progression of hepatitis B among regions, the patients’ medical records in HIS of Qinggang Healthcare Centre were reviewed to determine the onset rate of hepatitis B virus carriers to chronic hepatitis B patients. Since there were no patients with hepatitis B complications in the first round of screening and 11 complications in the second round, the low value in the guideline was adopted as the complication rate. In addition, an average of 10 years was used (triangulated) as survival time of hepatitis B complications based on proven research [13] and local specific characteristics. (Another factor that affected these flow rates in the model, but was not indicated in Fig. 2, is the aging of the population, which affects disease epidemic such as HBV onset rate and complication rate. For the sake of simplicity, the system dynamics model did not depict the additional factor as done by previous researchers [14]. However, by including the onset rate and the complication rate factors in hepatitis B, the model captured the consequences of age characteristics.)

The data were analysed using SPSS statistical analysis software (version 24). The prevalence rate of hepatitis B was defined as the number of HBsAg positive cases divided by the total number of residents screened for HBsAg during one round of screening (4 years). The incidence rate of hepatitis B (per 100 person-years) was defined as the number of HBsAg-negative people in the first round of screening but testing positive in the second round divided by the product of the number of people screened twice and the follow-up interval (years). The onset rate was measured in terms of the number of HBV carriers in Round 1 and progressing as chronic patients in Round 2 divided by the product of the number of people screened twice and the follow-up interval (years).

### Comparative experiments under different parameters of hepatitis B management strategy

To evaluate the effect of CCI HBV management on local HBV prevalence, comparative experiments for the system dynamics model could provide some answers. Here, we chose three of many possible scenarios for comparison with the baseline.

#### Enhanced vaccination of hepatitis B

Generally, the population born before 1992 was not administered with hepatitis B vaccine in Qinggang town. According to the screening results, few adults were HBsAb positive. Through simulation experiments, a full-aged HBV vaccination was conducted under different immune parameters.

#### Increased management of hepatitis B screening

The fraction of hepatitis B detection management with dynamic screening and regular follow-up could be summarised as a parameter. By comparing the different prevalence situation under different detection parameters, the impact of the diseases detecting measure on the local hepatitis B prevalence could be intuitively seen.

#### Increased management of hepatitis B normalised treatment

According to Chinese Hepatitis B Prevention Guidelines (2015 version), regular treatment of eligible hepatitis B patients could significantly improve the prognosis. At the moment, Interferon-a (INF-a) is rarely accepted because of the side effects. The major treatment for HBV patients in Qinggang area is normalised NAs antiviral therapy. The treatment parameter represents the effect of standardised treatment that meets the guidelines.

## Result

### The HBV screening and following-up of community cohorts

Table 1 presents the results of community HBV screening. So far, two rounds of HBV screening have been completed in Qinggang Town (2009–2012, 2013–2016). There were 12 228 people (5398 males and 6380 females) participating in HBV screening in Round 1 (2009–2012) with HBsAg positive prevalence of 8.5% (95% CI 8.0%–9.0%), consisting of 567 HBsAg positive males and 474 positive females, with the prevalence of 10.5% (95% CI 9.7%–11.3%) and 6.9% (95% CI 6.3%–7.5%), respectively. Of the 13 146 people tested in Round 2, 1171 people were HBsAg positive, with the prevalence of 8.9% (95% CI 8.4%–9.4%). Of the 6050 males tested in round 2, 661 were HBsAg positive, yielding a positive rate of 10.9% (95% CI 10.1%–11.7%). Of the 7096 females tested in round 2, 510 were HBsAg positive, with a positive rate of 7.2% (95% CI 6.6%–7.8%). The HBsAg positive rates varied widely among age groups. The positive rate of HBsAg in the 40–44 and 45–49 years was the highest (12.9% and 12.4% in Round 1, 13.3% and 13.4% in Round 2) during the two rounds of screening. The *P* value of the HBsAg prevalence was <0.05% between the two rounds, indicating that the local hepatitis B epidemic was stable.

**Table 1.** HBV prevalence in different genders and age groups in two rounds of screening

Groups	Sample size		No. of HBsAg positive		HBsAg positive rate(%)			
	Round 1	Round 2	Round 1	Round 2	Round 1	95% CI	Round 2	95% CI
Males	5398	6050	567	661	10.5	9.7–11.3	10.9	10.1–11.7
Females	6830	7096	474	510	6.9	6.3–7.5	7.2	6.6–7.8
0–24	2	159	1	9	–	–	5.7	2.0–9.3
25–29	13	275	3	37	–	–	13.5	9.4–17.5
30–34	87	444	18	65	20.7	1.2–29.4	14.6	11.3–17.9
35–39	145	663	14	84	9.7	4.8–14.5	12.7	10.1–15.2
40–44	419	828	54	110	12.9	9.7–16.1	13.3	11.0–15.6
45–49	1197	968	148	130	12.4	10.5–14.2	13.4	11.3–15.6
50–54	1466	1161	155	150	10.6	9.0–12.1	12.9	11.0–14.9
55–59	1475	1093	155	116	10.5	8.9–12.1	10.6	8.8–12.4
60–64	1842	1456	154	153	8.4	7.1–9.6	10.5	8.9–12.1
65–69	1637	1871	119	119	7.3	6.0–8.5	6.4	5.3–7.5
70–74	1119	1242	83	79	7.4	5.9–9.0	6.4	5.0–7.7
75–79	980	991	53	45	5.4	4.0–6.8	4.5	3.2–5.8
>80	1846	1995	84	74	4.6	3.6–5.5	3.7	2.9–4.5
All	12 228	13 146	1041	1171	8.5	8.0–9.0	8.9	8.4–9.4

By comparing the two rounds of screening, a total of 6377 people in the fixed cohort born before 1992 participated in both rounds of screening, including 470 HBsAg positive cases in Round 1 and 519 HBsAg positive cases in Round 2 (Table 2). By 2016, there were 49 people who were newly infected and the cohort interval of the follow-up was 4 years. As a result, HBV incidence rate in Qinggang Town was 0.192 per 100 person-years. By querying historical medical records, a total of 253 chronic hepatitis B patients were found in the first round and 389 in the second round with the cohort interval of the follow-up was 4 years. Thus, the hepatitis B onset rate was 0.533 per 100 person-years, which represented the progress of HBV carriers to CHB patients.

**Table 2.** HBV prevalence in different genders and age groups in fixed cohort

Groups	Group size	Round 1			Round 2		
		No. of HBsAg positive	HBsAg positive rate(%)	95% CI(%)	No. of HBsAg positive	HBsAg positive rate(%)	95% CI(%)
25–29	11	3	27.3	−4.1–58.7	3	27.3	−4.1–58.7
30–34	70	15	21.4	11.6–31.3	16	22.9	12.8–32.9
35–39	107	8	7.5	2.4–12.5	7	6.5	1.8–11.3
40–44	254	37	14.6	10.2–18.9	36	14.2	9.9–18.5
45–49	545	56	10.3	7.7–12.8	67	12.3	9.5–15.1
50–54	683	61	8.9	6.8–11.1	79	11.6	9.2–14.0
55–59	663	57	8.6	6.5–10.7	76	11.5	9.0–13.9
60–64	887	71	8.0	6.2–9.8	86	9.7	7.7–11.6
65–69	949	57	6.0	4.5–7.5	57	6.0	4.5–7.5
70–74	643	44	6.8	4.9–8.8	39	6.1	4.5–7.9
75–79	518	24	4.6	2.8–6.4	19	3.7	2.0–5.3
>80	1047	37	3.5	2.4–4.7	34	3.2	2.2–4.3
All	6377	470	7.4	6.7–8.0	519	8.1	7.5–8.8

### Output of hepatitis B epidemic situation modelling after 20 simulated years

**Fig. 3.**
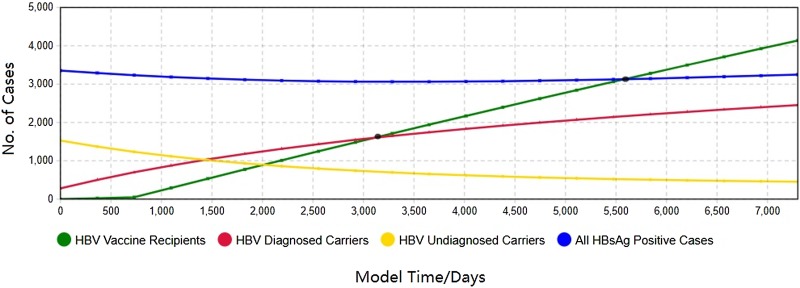
The 20-year HBV epidemic forecast in Qinggang, including diagnosed HBV carriers/patients/complications as well as immunised group.

To replicate the real-world situation, a simulated model of 41 197 agents was generated (long-stay population born before 1992 in Qinggang Town^1^), including 280 diagnosed carriers, 1529 undiagnosed carriers, 228 diagnosed CHB patients, 1245 undiagnosed patients, 11 diagnosed complications and 60 diagnosed complications (consistent with the fixed cohort in the end of Round 1 and 2 in 2016). The model then simulated the next two decades (7300 days). As a baseline scenario, it was assumed that there was no further change in the HBV incidence rate, the hepatitis B onset rate and the hepatitis B complication rate. The simulation parameters affecting the detection and treatment of HBV remained fixed through the period. Figure 3 presents selected out-put generated by simulating the model through a historical period starting in 2017 into the future through 2036. The number of uninfected people who received HBV vaccine had generally increased since 2019 (1% of the uninfected per year). The dynamic screening contributed towards finding undiagnosed HBV carriers/patients, who had gradually reduced over all these years. According to the outputs, there would be 4137 vaccine recipients, 2455 diagnosed carriers, 276 diagnosed chronic patients and 22 diagnosed complication patients, with 452 missed carriers, 41 missed chronic patients and 11 missed complication patients by the end of 2036. It should be noted that the model-output does not imply that its predictions will definitely happen in the future, but it does provide a rapid indicator of intervention implement.

Figure 3 depicts hepatitis B prevalence and disease progression from 2017 to 2036. Overall, the total number of HBV infections was slightly reduced in the local region. The implementation of dynamic screening led to an increase in the number of diagnosed hepatitis B carriers and at the same time, the number of undiagnosed carriers gradually decreased. Still, it would be unrealistic to find all the infections. According to output of Figure 3, if 1% of the uninfected people were immunised every year, the number of people with protective antibody would catch up with the number of diagnosed carriers after 2500 days (<7 years) of vaccine implementation and would catch up with all infected people after 4900 days(<14 years).

**Fig. 4.**
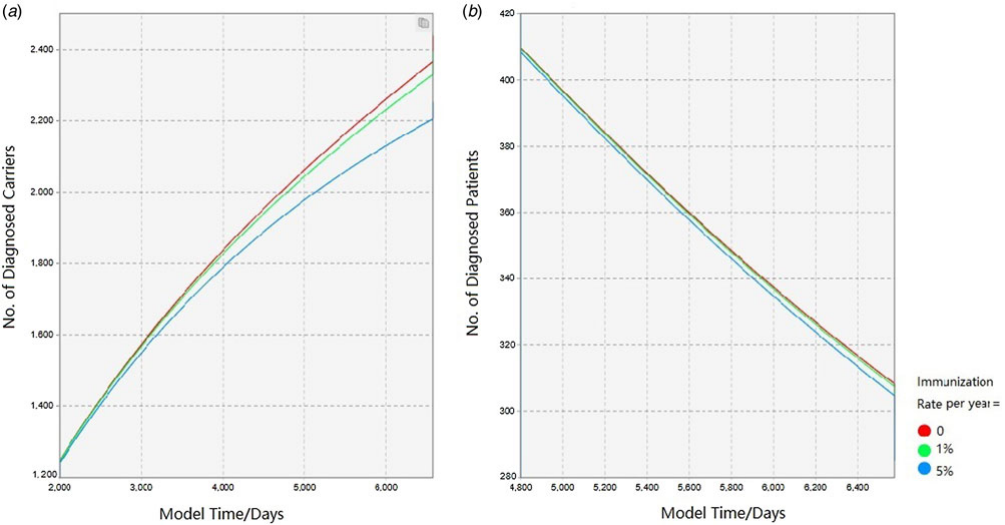
Comparison of epidemic forecast under different immunisation rates.

**Fig. 5.**
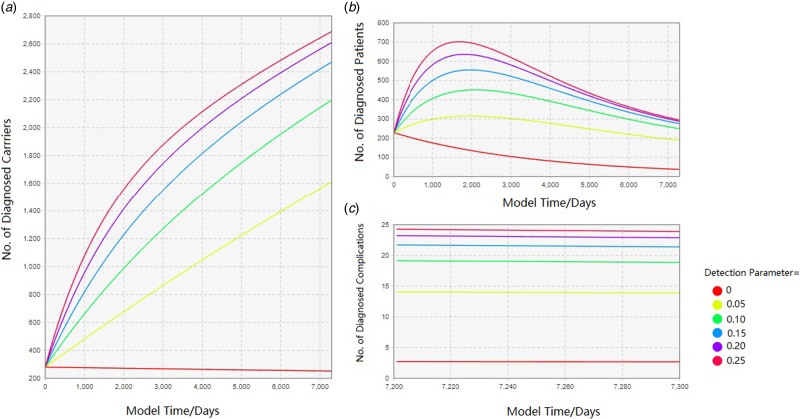
Comparison of epidemic forecast under different detection rates.

**Fig. 6.**
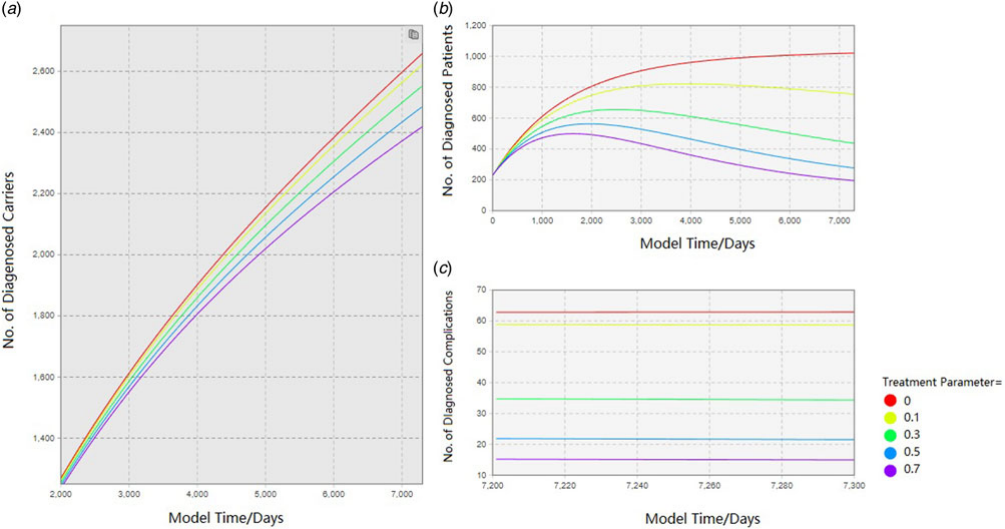
Comparison of epidemic forecast under different treatment rates.

The outputs of the three variables on the hepatitis B prevalence are shown in Figure 4–6. For clarity, the time of simulation started on 1 January 2017 and the *X*-axis and *Y*-axis range of some figures were narrowed. For each of the comparison test, these figures showed how interventions would alter the prevalence of local HBV status in the future.

#### Enhanced vaccination of hepatitis B

Based on the previous survey, it was assumed that hepatitis B vaccination rate per year in people born before 1992 was very low (<1‰ per year of the uninfecteds). The HBV immunisation project for all age groups in Qinggang is scheduled to begin in 2019 and this factor was considered in this study. Figure 4 indicates that, the intervention by full-aged HBV immunisation plan ultimately leads to a significant reduction in the number of diagnosed carriers in the future. The flow-out of the susceptible population, in turn, slows the number of HBV-related infections. The following is a comparison of four immunisation rates (0%, 1% and 5%/ year of the uninfected population, respectively). As the immunisation rate increases, the number of hepatitis B carriers and patients in the community consequently decreases. This strategy ensures that the establishment of the immune barrier contributes to the prevention and control of hepatitis B.

#### Increased management of hepatitis B screening

Due to the complexity of reality, it was impossible to make every resident in Qinggang participate in every round of screening. Thus, the screening strength was summarised into this parameter – the detection parameter. It was defined as the number of participants in one round of screening divided by the local long-staying residents. As a result of this intervention, more undiscovered HBV-related infections would be diagnosed. In the absence of hepatitis B screening, most of the local hepatitis B infections are hiding in the community. Once screening is started, the number of HBV-related cases increased, reflecting in diagnosed HBV carriers, patients and complications as well (Fig. 5). By summarising the screening effect as a parameter, it can be seen that as the screening intensity increases (0, 0.05, 0.10, 0.15, 0.20, 0.25), the growth rate of diagnosed carrier number in Figure 5a gradually become slower and the number of patients in Figure 5b increased synchronously. In a word, the growth rate of chronic hepatitis B patients gradually decreased in Qinggang town after most of the potential infections were diagnosed.

#### Increased management of hepatitis B normalised treatment

As a result of this intervention, the number of HBV infection declined. By summarising the effect into a treatment parameter, the HBV-related treatment for the infected people could reduce the number of hepatitis B carriers, hepatitis B patients and hepatitis B complications as well (Fig. 6, Treatment Parameter = 0, 0.1, 0.3, 0.5, 0.7). The decline of local HBV carriers is observed in the simulated contrast experiment, which reduced the number of patients and complications. Besides, the normalised treatment slows down the disease progress and reduces the number of onset patients and complications. The number of patients with complications would be more pronounced at the end of the simulation due to many reasons including survival time of complication patients. By comparing the prevalence rate under different treatment parameters, it could be seen that as the intensity of treatment implementation increases, the number of HBV infections gradually decreases. Similarly, due to the actual situation, it is unrealistic to perform comprehensive treatment for all patients and the treatment cannot be effective for all patients. This parameter is also unlikely to reach 100%.

## Discussion

Public health research, in China and elsewhere, is increasingly focusing on policy implement. To improve efficiency, it is urgent to develop a tool to fully identify those healthcare policies and interventions to avoid the waste of time and manpower. As model simulation thriving in many fields, it is imperative to combine the strength of field research and simulation experiment in disease prevention. After two rounds of screening and follow-up for long-staying residents by 2016, some HBV epidemiological features in Qinggang were obtained to accurately predict local HBV prevalence status. In particular, this study could improve understanding of several aspects in HBV prevention and control in the community.

First, the study presented an example to identifying the interventions of diseases control. With the key data that fit local HBV epidemiological characteristics being collected, the regional prevalence in the future was predictable based on simulation. By summarising the implementation effects of three interventions into key parameters, we could compare the prevalence status under different intensity. This method not only has a reference value for hepatitis B control but holds significance for the prevention and control of other infectious and chronic diseases. For example, the management of diabetes and hypertension can be combined with field investigation and computer modelling to utilise field investigation for reliable data and dynamic modelling for prediction and reference, conversely. Besides, this model can be used for health economics research. With further cost calculation function, the cost of various policies during various disease stages can be calculated dynamically.

Second, the role of the immune barrier in the prevention and control of hepatitis B was quantified and visualised. Many studies have demonstrated the effect of the immune barrier on the disease prevention and treatment [6, 7, 15]. From the perspective of disease control, it holds greater meaning to maintain a healthy community rather than treat the infected patient in the hospital. However, HBV vaccination among adults in China is not yet active [2] and the coverage of adult HBV vaccination is very low. This fact, combined with the highest HBV prevalence in the age group of 35–39 was 9.93 (95% CI 8.93%–9.73%) [7], is leading to a crisis that adults born before 1992 could be more vulnerable to hepatitis B virus. According to the 2010 National Population Census, there were one billion adults born before 1990 in 2010 [16]. To use vaccination as the HBV control strategy, public health policies would target healthy adults in rural areas. According to this study, the increase in the number of vaccinated people also slowed the spread of hepatitis B, which was reflected in the decreasing slope of the curves representing hepatitis B infected people. On the other hand, full-aged HBV immunisation allows more adults to receive hepatitis B vaccine. As the curve gradually become flat, the establishment of HBV immune barrier results in a lower incidence of hepatitis B infection and a lower onset rate of local chronic hepatitis B patients. However, the number of healthcare providers in Qinggang is limited. If the number of people needing to be immunised is exceeding the load capacity of the local community healthcare system, the decline of hepatitis B carriers would not perform as expected. After considering the community workload and immune effect, we recommend 1%–5% of the uninfecteds as the immunisation rate per year. In other words, they need to vaccinate about 400–2000 people per year, which is decreasing year by year. The intensity of the immunisation should be fully adapted to the capability of the local community healthcare provider.

Third, the intervention for HBV infection cases needs to be diverse. After hepatitis B dynamic screening in the community, more undiagnosed HBV cases are found and included in the follow-up cohort. This explains the increase in diagnosed HBV-infection groups caused by dynamic screening. This increase was not due to a spurt growth in hepatitis B new infections. Rather, many patients were not aware of the necessity of regular medical examinations and have long been unknown of their disease conditions without screening. In a word, improving the screening capacity of the local community helps to fundamentally minimise the source of infection. Meanwhile, the usefulness of detection and treatment parameters may contribute to evaluate the efficiency of community interventions. By comparing predictions and realities, the performance of local hepatitis B screening can be assessed. Nomograms are in preparation for intervention effect evaluation in screening and treatment, respectively. Besides, social work organisation and patient support teams also play a role in improving patient compliance in the community.

Hepatitis B infection remains a serious public health problem worldwide [3, 17–19]. In 2017, the reported cases of notifiable infectious diseases in China were 7 030 879, of which the number of HBV cases was 1 001 952 including 425 deaths [20]. In other words, hepatitis B accounted for 7.02% among all reported infectious cases in 2017 in China. According to recent research, the prevalence of HBsAg in China in 2016 was estimated to be 6.1% (95% CI 5.5%–6.9%) and the number of HBsAg-positive people was approximately 86.607 million. The number of patients needing treatment was 32.315 million, the actual number of patients treated was 3.5 million, the treatment rate was 11% and the number of diagnoses was 16.058 million with the diagnose rate of only 19% in 2016 [6]. In Zhejiang province, it is reported that the HBsAg carrier rate varied from 5% to 13% [21]. In the face of the low diagnostic rate and low treatment rate of HBV infection, a more diversified approach is needed to deal with this public health threat. To explore an efficient and affordable strategy of HBV control, CCI HBV demonstration area project was initiated in 2009. Combined with the progress of Chinese New Medical Reform, the project was committed to integrating community medical centres and clinics, focusing on community-level intervention: (1) it achieved HBV dynamic screening for residents through community medical gridding and helped to locate the source of infection in community; (2) it provided the normalised treatment for hepatitis B infections promptly, to slow the progression of the disease and reduce the complications occurrence; (3) the full population coverage (including adults, teenagers, children and new-borns) of hepatitis B vaccination in the community will start from 2019 and its effect will be observed.

System dynamics modelling of high-level abstraction can be used to structure predictive model of hepatitis B. The computer modelling actually creates a sandbox, in which many policies, interventions and capacities can test their efficacy and efficiency as well. Without making large sunk-cost for uncertain outcomes, it is possible to have the evidence-based performance for the disease interventions based on visualisation and dynamics. Nowadays, there are already many modelling solutions for medical applications. Instead of applying average data to a large group/region, the most obvious feature of this study is the implementation of specific epidemiological simulation in the corresponding community. For other regions, the data may be different, but the method is consistent.

Besides such extensions, more work remains in epidemiological data collection and analysis. Based on reality, the aging of the local population in the cohort affected the spread and development of the disease in many aspects. The impact of this demographic imbalance on the model is not known and there is a need for further expansion of the breadth of data collection to obtain more information which is consistent with the reality. A medium abstract level simulation needs to be elaborated to a specific internal situation of HBV infection group, focusing on individual changes within the population. Moreover, it is necessary to encourage more people to participate in local hepatitis B screening to improve the effectiveness and accuracy of interventions. In summary, there is a need to continue the multi-dimensional management of hepatitis B and get closer-to-real information as much as possible to improve the prediction accuracy of the simulation. In conclusion, this simulation modelling and experimentation could help public health policymakers to better evaluate the effects of intervention in infectious diseases as well as other chronic diseases.

## Footnotes

1 This data came from Yuhuan City Statistics Bureau.
